# Chemical Genetics Screen of EVP4593 Sensitivity in Budding Yeast Identifies Effects on Mitochondrial Structure and Function

**DOI:** 10.17912/micropub.biology.000806

**Published:** 2023-04-25

**Authors:** Lexie Hiestand, Stella Shen, Willough Sloan, Hamid Nasiri, Dana Lashley, Oliver Kerscher

**Affiliations:** 1 Biology, William & Mary, Williamsburg, Virginia, United States; 2 Chemistry, William & Mary, Williamsburg, Virginia, United States; 3 Department of Cellular Microbiology, University Hohenheim, Stuttgart, Germany

## Abstract

Mitochondria are essential eukaryotic organelles. Mitochondrial dysfunction can lead to mitochondrial myopathies and may contribute to neurodegenerative diseases, cancer, and diabetes. EVP4593, a 6-aminoquinazoline derivative with therapeutic potential, has been shown to inhibit NADH–ubiquinone oxidoreductase (Complex I) of the mitochondrial electron transport chain, causing the release of reactive oxygen species (ROS) and a reduction in ATP synthesis. In isolated mitochondria, EVP4593 inhibits respiration in the nanomolar range (IC _50_ = 14-25 nM). However, other EVP4593-specific effects on biological processes have also been described. Consistent with an effect on mitochondrial function in budding yeast, we find that EVP4593 [>25µM] induces a pronounced growth defect when wildtype cells are grown on a non-fermentable carbon source. This sensitivity to EVP4593 is exacerbated by deletion of *PDR5* , an ABC transporter that confers multidrug resistance. To better understand the cellular pathways and processes affected by EVP4593, we conducted a genome-wide chemical genetics screen of the yeast knockout collection. The objective was to identify yeast gene deletion strains that exhibit growth defects when subjected to a sublethal concentration of EVP4593 [15µM]. Our screen identified 21 yeast genes that are required for resistance to 15µM EVP4593 in glycerol-containing media. The genes identified in our screen are functionally involved in several distinct categories including mitochondrial structure and function, translational regulation and nutritional sensing, cellular stress response and detoxification. Additionally, we identified cellular phenotypes associated with the exposure to EVP4593, including changes in mitochondrial structure. In conclusion, our study represents the first genome-wide screen in yeast to identify the genetic pathways and cell-protective mechanisms involved in EVP4593 resistance and reveals that this small molecule inhibitor affects both mitochondrial structure and function.

**Figure 1. EVP4593-sensitivity screen of the yeast deletion library and analysis of its functional effects:**
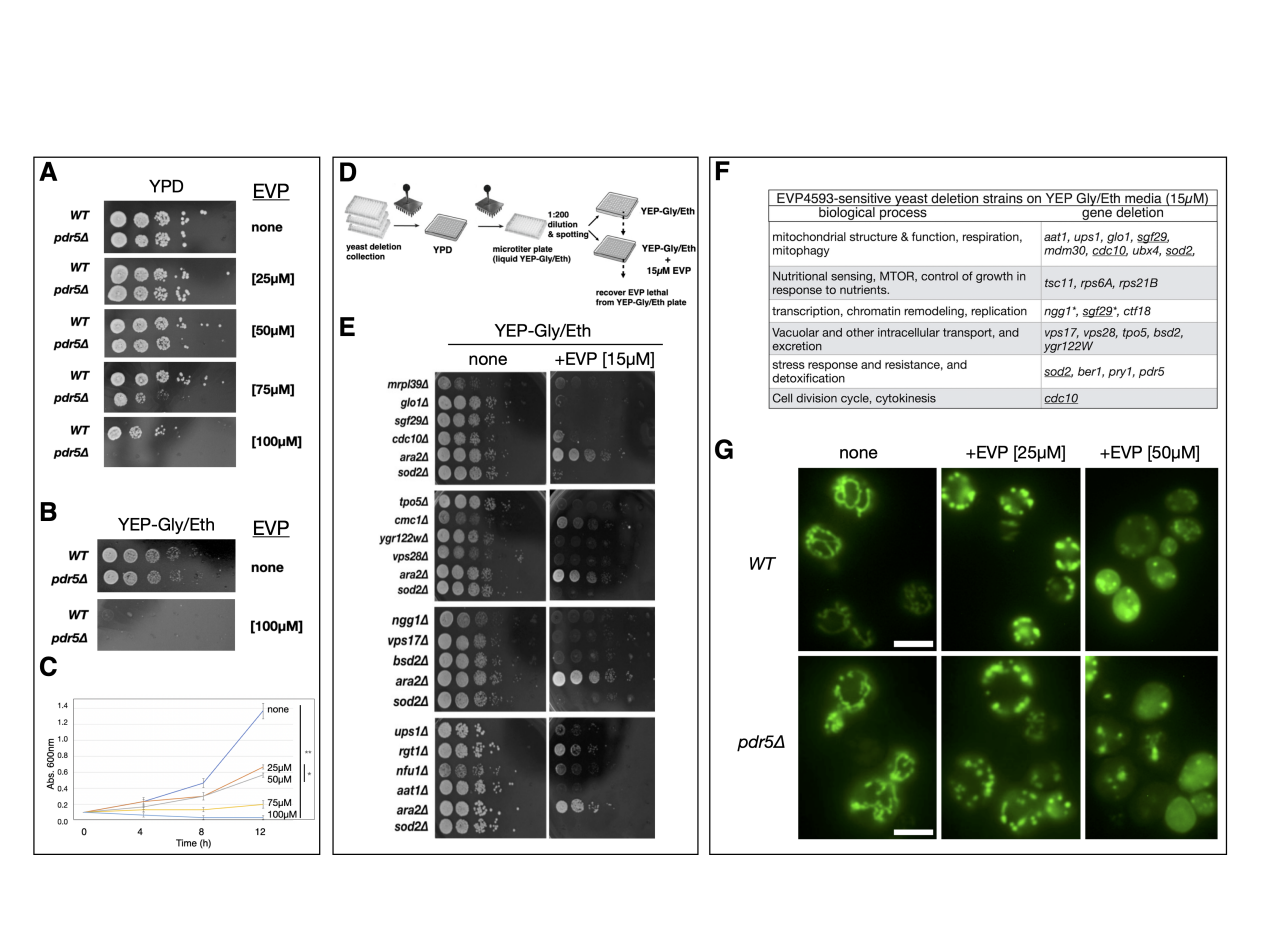
( **A)** Serial dilution growth assay of WT and *pdr5∆* strains on YPD media only (none) or YPD media with indicated concentration of EVP4593 (µM). ( **B)** Serial dilution growth assay of WT and *pdr5∆* strains grown on YEP Gly/Eth media only (none) or in the presence of 100μM EVP4593 ( **C)** Growth Assay of WT cells in liquid YEP Gly/Eth media only (none) or with addition of the indicated concentration of EVP4593. Absorbance measurements (Abs 600nM) and corresponding time (hours) after EVP-treatment (µM) as indicated. Statistical analysis by ANOVA and comparative significance was determined by a Tukey test. At 12 hours (NS:P ≤ 0.01**, P > 0.05*) ( **D)** Schematic of the primary EVP-sensitivity screen: >5100 strains of the yeast deletion library were grown up in 96-well plates, diluted, and spotted to YEP Gly/Eth plates with or without 15μM EVP-4593 to identify sensitive deletion mutants. **(E)** Serial dilution growth assay of select knockout strains identified in the primary screen with EVP-sensitive ( *sod2∆* ) and EVP-resistant ( *ara2∆* ) controls on YEP Gly/Eth plates with or without 15μM EVP-4593. ( **F** ) Final table of 21 confirmed EVP4593 sensitive yeast deletion strains identified in the EVP-sensitivity screen. Individual gene deletions and corresponding biological process are indicated. Deletions in more than 1 category (e.g. *sod2* ) are underlined, * indicates Sgf29 and Ngg1 are both components of the HAT module of the SAGA complex (Hemlinger and Tora, 2017). *pdr5∆* shows increased sensitivity to EVP459 but was not identified in the primary screen. ( **G** ) Mitochondrial morphology in WT and *pdr5∆* strains grown in YEP Gly/Eth media only (-) or in the presence of 25 and 50μM EVP4593 after 5 hours at 30°C, as indicated. Scale bar 5μM.

## Description

We conducted a genome-wide chemical genetics screen of the yeast knockout collection to identify non-essential genes that sensitize yeast cells to EVP4593 treatment. EVP4593, a quinazoline derivative with a hydrophobic phenoxyphenyl-substituent, is an established small molecule inhibitor of complex I of the mitochondrial electron transport chain (MCI), inhibiting respiration of isolated mitochondria in the nanomolar range (IC _50_ = 14-25 nM) (Krishnathas et al. 2017) . In cultured cancer cells 1µM EVP4593 inhibits cellular respiration significantly, reducing viability under glucose starvation conditions. (Marciano et al. 2019, Luise et al. 2022) . EVP4593 has also been reported to act as a potent inhibitor of NF-κB transcriptional activation [IC _50_ 2 nM], which exhibits anti-inflammatory properties in mice and neuroprotective effects in a *Drosophila* model of Huntington’s disease (Tobe et al. 2003a, Tobe et al. 2003b, Wu et al. 2007, Vigont et al. 2018) . How EVP4593 induces these effects remains unknown and, therefore, a genome-wide EVP4593-sensitivity screen of the yeast knockout collection may provide insights into the underlying genetic and cell-protective pathways.

To determine the effect of EVP4593 on *Saccharomyces cerevisiae* growth, we initially spotted cells on media plates containing 2% glucose (a fermentable carbon source) and varying concentrations of EVP4593 ( **A** ). First, we tested the EVP4593 sensitivity of isogenic BY4741 wildtype (WT) and *pdr5::KANMX4* ( *pdr5∆* ) deletion strains. We reasoned that deletion of *PDR5* , an ABC transporter that confers multidrug resistance and interacts with lipophilic substrates (Wagner et al. 2019) , would render cells more sensitive to EVP4593 which carries a hydrophobic phenoxy-substituent (Krishnathas et al. 2017) . Indeed, on glucose-containing media (YPD), *pdr5∆* cells showed a severe growth defect at 75µM and failed to grow completely at 100µM EVP4593 **(A)** . In contrast WT cells survived up to 100µM EVP4593 on glucose-containing media. These data suggests that EVP4593 may be a substrate of the multi-drug transporter *pdr5* and that its inhibitory effect on yeast grown on a fermentable carbon source (glucose) is limited, although not absent.

Since EVP4593 is an established MCI inhibitor, we next tested its effect on yeast cell growth on media plates containing 2% glycerol and ethanol (YEP Gly/Eth) as non-fermentable carbon sources. Yeast growth on YEP Gly/Eth requires the functional electron transport chain (ETC) of mitochondria. Both WT and *pdr5∆* cells were able to grow on YEP Gly/Eth media indicating respiration competence. However, consistent with inhibition of the ETC at MCI, both WT and *pdr5∆* cells completely failed to grow on YEP Gly/Eth media containing 100µm EVP4593 **(B)** . We quantitated the effect of EVP4593 on WT cells using a growth assay in liquid YEP Gly/Eth media with various concentrations of EVP4593 (0, 25, 50, 75, or 100µM). After 12 hours, growth in all concentrations of EVP4593 was significantly reduced in comparison to untreated cells (p < 0.01) **(C)** . These results indicate a significant effect of EVP4593 on WT yeast cell growth above 25µM when cells depend on their mitochondria for growth. This finding correlates with the observed growth defect of EVP-treated cancer cells grown under glucose starvation (Marciano et al. 2019) .

The goal of this project was to identify the genetic pathways and cell-protective mechanisms involved in EVP resistance. Based on the above growth assay with WT cells we screened the entire MATa yeast knockout collection (>5100 strains) on YEP Gly/Eth media for EVP-sensitive strains (Giaever et al., 2002) . For our primary screen we used a sublethal concentration of 15µM EVP added to the media ( **D** ). We recovered about 100 EVP-sensitive candidate strains from the primary screen and re-tested them on YEP Gly/Eth media with and without 15µM EVP, using a sensitive cell dilution and spotting assay ( **E** ). Using this approach, our screen ultimately netted 21 deletion strains that grew robustly on YEP Gly/Eth media but consistently showed greatly reduced or no growth on the same media with 15µM EVP4593 (referred to as EVP-sensitivity) ( **F** ). Functionally, the genes identified in our screen fell into 5 main categories: Mitochondrial structure and function (eight genes), nutritional sensing (three genes), cellular stress response (four genes), chromatin organization and transcriptional regulation (three genes), vacuolar and other intracellular transport (five genes).

Highlights from our screen include the *sod2∆* mutant which is highly sensitive to EVP4593 and also serves as a positive control for the secondary growth assays ( **E, F** ). *SOD2* encodes the yeast mitochondrial superoxide dismutase and yeast strains carrying a *sod2∆* deletion are very sensitive to oxidative stress. The effect of EVP4593 on the *sod2∆* mutant is consistent with the prior finding that inhibition of MCI by EVP4593 results in the release of reactive oxygen species (ROS) (Krishnathas et al. 2017) . About one third of the EVP4593 sensitivity genes (7/21) identified by us affect mitochondrial respiration ( *AAT1, UPS1, GLO1, SGF29, MDM30, UBX4, SOD2* ), localize to mitochondria or affect their structure/assembly ( *AAT1, UPS1, MDM30, SOD2, CDC10* ), or have abnormal mitochondrial genome maintenance (UBX4) (Cherry et al. 2012, Barve et al. 2018) . The next category includes 5 genes involved in vacuolar and intracellular transport, and excretion ( *VPS17, VPS28, TPO5, BSD2, YGR122W* ). Deletions of these genes are all reported to have abnormal vacuolar morphology. As vacuoles in yeast are involved in degradation of damaged organelles/proteins and respond to nutrient stress, this may reflect the cellular need to respond to EVP-damaged mitochondria (Li and Kane 2009, Stauffer and Powers 2017) . Related, we also identified 4 genes we categorized in stress response, stress resistance, and cellular detoxification ( *SOD2, BER1, PRY1, PDR5* ). *PDR5* (Fig. A,B) and the fatty acid and sterol binding protein *PRY1* are both involved in the removal of hydrophobic compounds, such as EVP4593, from the cell (Cherry et al., 2012) . Identification of *SOD2* , the mitochondrial superoxide dismutase suggests that at least part of EVP4593’s effect is due to the increase of ROS that damage proteins, DNA, and lipids. It is not entirely clear why we also identified *BER1* , a gene involved in microtubule stability (Fiechter et al. 2008) . However, another mitochondrial MCI inhibitor, rotenone, interferes with microtubule assembly (Heinz et al. 2017) . Finally, we identified several genes broadly categorized in nutritional sensing, transcription, and translational control. As EVP4593 inhibits mitochondrial respiration, nutritional reprogramming is likely a major requirement for cell survival, involving the interplay of TOR-mediated nutritional sensing ( *TSC11* ) and chromatin remodeling ( *SGF29, NGG1* ). In summary our data place into focus the effect of EVP4593 on mitochondrial respiration combined with a need for detoxification and metabolic reprogramming after exposure to this small molecule inhibitor.

Since our screen uncovered several genes with a role in mitochondrial structure and function, we decided to visualize yeast mitochondria after EVP4593 treatment ( **F** ). We transformed both WT and *pdr5∆* cells with a plasmid expressing a mitochondrial matrix-targeted red fluorescent protein (su9-RFP). Log-phase cells with labeled mitochondria were treated with varying concentrations of EVP4593 (0, 25, 50 µM) for 5 hours. As expected, imaging mitochondria in untreated yeast cells revealed a net-like reticulum with little or no staining in the cytoplasm, consistent with intact and functional mitochondria. In contrast, cells treated with 25µl EVP4593 were devoid of elongated organelles, revealing mitochondria fragments. Increase of EVP4593 (50µM) resulted in further disruption of mitochondrial fragments, an increase in cytoplasmic straining, or diffuse staining in some cells ( **F** ). Nevertheless, after this acute treatment cells remained viable on both glucose and non-fermentable media when the EVP treatment was removed. This indicates that the observed morphology changes were not staining artifacts of dead or dying cells but represent an effect of EVP4593 on mitochondrial morphology in yeast, which has not previously been described. We did not observe any quantifiable differences between *pdr5∆* and WT cells at the concentrations tested. However, we predict that *pdr5∆* strains may show mitochondrial fragmentation at lower EVP4593 concentrations that do not affect WT cell. Mitochondrial fragmentation itself may be due to the production of ROS, as has been attributed to the MCI inhibitor rotenone and the complex II inhibitor 3-NP in neurons and epidermal cells, respectively (Liot et al. 2009, Fu et al. 2020, Li et al. 2003) . Generally, fragmentation negatively affects mitochondrial metabolism and the maintenance of mitochondrial DNA. Notably, mitochondrial structure and dynamics are often altered in cancer and neurodegenerative disease (Corrado, Scorrano and Campello 2012) .

In summary, our screen identified genes required for the resistance to EVP4593, including those that affect mitochondrial structure and function. Combined with the effect of EVP4593 on mitochondrial morphology described above, our study suggests that mitochondria (and presumably the ETC) are the primary target of this quinazoline derivative in yeast. Based on our data, we hypothesize that due to the inhibition of mitochondrial respiration and the ensuing production of ROS, EVP4593 treatment triggers metabolic reprogramming and several stress response pathways. Future studies in yeast may elucidate additional cellular targets and effects attributed to EVP4593 and its derivatives (Grekhnev et al. 2022) .

## Methods

**Yeast cell growth, EVP treatment, and microscopy** : Standard yeast genetic techniques and media were used (Rose et al., 1990). We quantitated the effect of EVP4593 grown WT cells using a growth assay in liquid YEP Gly/Eth media (Yeast extract, peptone, 2% glycerol and ethanol). Briefly, WT cells were grown to logarithmic phase and then diluted in YEP Gly/Eth with various concentrations of EVP4593 (0, 25, 50, 75, or 100µM). OD600 measurements of each culture were taken every 4 hours. After 12 hours, growth in all concentrations of EVP4593 was significantly reduced in comparison to untreated cells (p < 0.01). Statistical analysis by ANOVA and comparative significance was determined by a Tukey test. At 12 hours (NS:P ≤ 0.01**, P > 0.05*). For yeast cell microscopy, su9-RFP/URA3 plasmid (pFL8, a gift from Prof. Yasushi Tamura, Yamagata University, Japan) was transformed in WT or *pdr5∆* cells, transformants were selected on SD-URA media, grown overnight in YEP Gly/Eth media with or without EVP4593. Images of live cells with stained mitochondria were collected using a Zeiss Axioskop microscope (Carl Zeiss USA, Thornwood, NY, USA) fitted with a QImaging Retiga™ digital camera (QImaging, Surrey, BC, Canada), i-Vision-Mac software (BioVision Technologies, Exton, PA, USA) and a Uniblitz shutter assembly system (Vincent Associates/UNIBLITZ, Rochester, NY, USA), with the appropriate chroma filter set.

**EVP-sensitivity screen** : Individual 96-well plates of the yeast knockout collection were thawed, and cells were spotted on YPD media. Once colonies had formed, individual strains were diluted in 96-well plates with liquid YEP Gly/Eth media for 48 hours. Cells in each well were then diluted 1:200 in water before spotting on YEP Gly/Eth media with (or without) 15µM EVP4593. EVP4593 lethal strains were recovered from the YEP Gly/Eth plate after 3-5 days and retested. All strains identified in this primary screen were also recovered from the original library plate for secondary testing on EVP4593 plates. This approach led to a final list of 21 yeast strains that reproducibly show EVP4593 sensitivity. In our assays a EVP4593-sensitive *sod2∆* strain (carrying a deletion of the mitochondrial manganese superoxide dismutase) and a EVP4593-resistant *ara2∆* strain (carrying a deletion of the NAD-dependent arabinose dehydrogenase) served as positive and negative controls, respectively. The *ara2∆* strain was included as a negative control because it grew robustly on Gly/Eth media with a sub-lethal dose of EVP4593 [15µM] and because it was marked with the G418-resistance marker, *KANMX4* . Therefore, we are adding the caveat that data shown in Fig. 1E lacks direct comparison to an isogenic wildtype control as in Fig 1A, B, C. Biological Process information was assigned using information from www.yeastgenome.org (Cherry et al., 2012) and Panther GO slim (pantherdb.org).

## Reagents

EVP4593 for the screen was purchased from Selleckchem and MedChemExpress (Catalog HY-13812).
